# Proteomics of Toxigenic Corynebacteria

**DOI:** 10.3390/proteomes12010002

**Published:** 2023-12-30

**Authors:** Andreas Burkovski

**Affiliations:** Microbiology Division, Friedrich-Alexander-Universität Erlangen-Nürnberg, 91058 Erlangen, Germany; andreas.burkovski@fau.de; Tel.: +49-91318528086

**Keywords:** caseous lymphadenitis, *Corynebacterium*, diphtheria, exoproteome, proteome complexity, reverse vaccinology, ulcerative lymphangitis, zoonosis

## Abstract

Within the genus *Corynebacterium*, six species are potential carriers of the *tox* gene, which encodes the highly potent diphtheria exotoxin: *Corynebacterium diphtheriae*, *Corynebacterium belfantii*, *Corynebacterium rouxii*, *Corynebacterium ulcerans*, *Corynebacterium pseudotuberculosis* and *Corynebacterium silvaticum.* Based on their potential to infect different host species and cause either human infections, zoonotic diseases or infections of economically important animals, these bacteria are of high scientific and economic interest and different research groups have carried out proteome analyses. These showed that especially the combination of MS-based proteomics with bioinformatic tools helped significantly to elucidate the functional aspects of corynebacterial genomes and to handle the genome and proteome complexity. The combination of proteomic and bioinformatic approaches was also used to discover new vaccine and drug targets. In addition, matrix-assisted laser desorption/ionization time-of-flight mass spectrometry has been established as a fast and precise tool for the identification of these bacteria.

## 1. Relevance and Properties of Toxigenic Corynebacteria

Corynebacteria belong to the phylum *Actinobacteria*, Gram-positive bacteria with a high G+C DNA content [1]. Within this phylum, they form the CMNR group together with the genera *Mycobacterium*, *Nocardia* and *Rhodococcus* based on a complex mycolic acids-containing cell wall structure, which is distinctive of these four genera [2]. To date, 158 taxonomically valid *Corynebacterium* species are described [3]. More than half of these were isolated from human and animal material, indicating the considerable medical and veterinary importance of the genus [3]. This is especially true for the group of toxigenic corynebacteria initially formed by *Corynebacterium diphtheriae*, *Corynebacterium ulcerans* and *Corynebacterium pseudotuberculosis* [4]. These species can be lysogenized by *tox* gene-encoding corynebacteriophages and subsequently express and secrete the potent diphtheria exotoxin [5]. The recently described new species *Corynebacterium belfantii*, *Corynebacterium rouxii* and *Corynebacterium silvaticum* are also potential carriers of the diphtheria toxin-encoding *tox* gene and may therefore be included in this group [6,7].

### 1.1. Corynebacterium diphtheriae

*C. diphtheriae* is the etiological agent of diphtheria, a putatively fatal infection of the upper respiratory tract characterized by sore throat, fever and pseudomembrane formation [8]. In addition to respiratory diphtheria, *C. diphtheriae* can cause skin lesions and systemic infections such as arthritis, bacteremia and endocarditis. While the diphtheria toxin is responsible for the often fatal outcome of the infection, additional virulence factors influence adhesion and invasion as well as the survival of the bacteria in macrophages [9].

Almost twenty years ago, Hansmeier and co-workers [10] started a proteomic approach to identifying the secreted proteins of *C. diphtheriae* based on the idea that these are especially important for pathogen–host interaction. Using protein separation via two-dimensional gel electrophoresis to separate the proteins by means of the isoelectric point and apparent molecular mass, tryptic digestion of excised spots and peptide mass fingerprint analysis, 85 different secreted proteins were identified in the extracellular and cell surface proteome fraction, including different putative virulence factors. Partially based on this work, these proteins were further characterized in independent studies, e.g., the neuraminidase/exo-alpha sialidase NanH [11], the invasion-associated protein DIP1281 [12,13] or the fimbrial protein-associated sortase SrtC [14].

To characterize the function of such virulence factors, often host–pathogen interaction studies using murine or human cell lines are carried out. For this kind of experiment, infection has to be carried out in cell culture media. To address the question of the putative influence of the cell culture media and their respective components, the *C. diphtheriae* strain ISS3319 was cultivated in a standard bacteriology medium, cell culture medium and fetal calf serum. The proteins were alkylated, digested and purified using C18 stage tips. Subsequent mass spectrometric analyses in combination with label-free protein quantification using the total protein approach (TPA) method [15] indicated the influence of the growth medium on the cell envelope and an increase in pathogenicity when bacteria were grown in the cell culture medium [15]. Pathogenicity-connected proteins, such as the multifunctional protein DIP0733, the conserved hypothetical protein DIP1546 and the resuscitation promoting factor RpfB (DIP0874), were induced in cell culture medium or serum even without host cell contact. Furthermore, the cell culture conditions led to the preadaptation of *C. diphtheriae* to host cell contact via the induction of iron starvation, cell envelope changes as well as oxidative and nitrosative stress response mechanisms [15].

Other proteome studies led to the identification of regulatory proteins, which are possibly involved in the coordination and control of NO stress in *C. diphtheriae*, i.e., DtxR and DIP1512 [16], and defined the role of S-mycothiolation in redox control under oxidative stress [17]. Using shotgun-proteomics, 26 S-mycothiolated proteins of the *C. diphtheriae* strain DSM43989 were identified in response to hypochlorite treatment. Post-translational thiol-modifications were identified by searching all the MS/MS spectra against the *C. diphtheriae* target–decoy protein sequence database using the Sorcerer-SEQUEST program package [17]. In addition to the oxidative stress response, these were involved in pathways with central physiological functions like energy metabolism, amino acid and nucleotide biosynthesis, with protein synthesis indicating an important function of S-mycothiolation in the survival of redox stress in *C. diphtheriae* [17].

In contrast to the described in vitro studies, which focused on single strains, a number of in silico proteome studies were carried out, which are especially suited to address the proteome complexity of *C. diphtheriae*. In this organism, a restricted number of core genes are accompanied by strain-specific accessory genes often acquired via horizontal gene transfer. Already a first pangenome study with 13 isolates of this pathogen showed a number of gain and loss of function processes, and the authors concluded that these genome variations may reflect a strategy of *C. diphtheriae* to establish different host–pathogen interactions such as respiratory or systemic infections [18]. In frame of the study, from the 13 genomes, a core genome comprising 1632 protein-encoding genes and a pangenome of 4786 genes was established with an average of 65 unique new genes per genome sequence analyzed. In a similar study based on 24 genome sequences, a pan-genome comprising 3989 protein-coding sequences, including 1625 core genes and 2364 accessory genes, was identified. Most pathogenicity islands predicted in this study encode subunits of adhesive pili, which may play a role in the adhesion of *C. diphtheriae* to different host cells. In fact, Sangal and co-workers found that the adherence and invasion capacity of *C. diphtheriae* isolates correlate with the predicted membrane-associated and secreted proteome and that the proteome variability and complexity are mainly based on hypothetical proteins [19]. Differences in the ability of *C. diphtheriae* strains to adhere to host cells correlate especially with the number and organization of the pilus gene clusters. Also, the number of genes encoding surface proteins with LPXTG motifs differed between the investigated strains and may result in variations in their adhesive properties [19].

In addition to the described characterization of virulence factors, signaling pathways and genome variability, proteomics approaches were applied to discover new treatment and prevention methods. In silico approaches focused on strategies to identify essential proteins as targets to develop new preventive vaccines or drug targets. Already in 2008, Dass and Deepika showed that the scanning of immunologically relevant regions of bacterial protein sequences, i.e., membrane and membrane-associated proteins, is suitable for the identification of specific HLA-binding peptides for the design of putative vaccine candidates [20]. In this study, 355 membrane or membrane-associated proteins, fimbrial subunits and surface-anchored proteins were selected from the annotated protein sequences and the antigenic subsequences of these proteins were identified, which were further used for the prediction of MHC class II HLA DRB1 allele binding regions. A total of 30 short peptides of membrane proteins were identified using this approach [20].

In an integrative in silico approach for therapeutic target identification, Jamal and co-workers used 13 genome sequences to establish a core genome of 423 genes. Using subtractive proteomics and modelomics approaches, 23 essential genes were identified. Eight of these were considered as therapeutic targets based on the absence of host homologs [21] (Table 1) and three were in addition identified in a subsequent modeling study based on the same genome set [22] (Table 1). Most of these targets identified via in silico approaches were verified. Transposon Directed Insertion Sequencing (TraDIS) of a high-density transposon mutant pool indicated the corresponding genes as essential when *C. diphtheriae* was grown in broth [23]. Today, none of these putative drug and vaccine targets are tested in clinical trials, since antibiotic resistances are rare in *C. diphtheriae* and the toxoid vaccine directed against the diphtheria toxin has been successfully applied for more than 100 years.

For more information about the toxoid production process and the reason for a drift toward infections with non-toxigenic strains [24], preparations of commercially available diphtheria toxoid vaccine were studied via proteome analyses [25]. For this purpose, formaldehyde crosslinking used to inactivate the fatal diphtheria toxin and produce the safe toxoid was reversed by means of heat treatment. Proteins were digested using a filter-aided sample preparation protocol and mass spectrometric analyses were carried out, followed by label-free quantification [25]. As expected, the inactivated diphtheria toxin is, with approximately four-fifths, the main component of vaccine preparations from Brazil, Bulgaria, Germany, India and Russia. As shown in this study, vaccination protects against the fatal diphtheria exotoxin expressed by *C. diphtheriae* and also by *C. ulcerans*. However, a significant number of additional *C. diphtheriae* proteins was found in all the tested vaccines, summing up to about 20% of the protein content. In total, 665 different proteins were identified in the tested vaccines, varying between 130 and 436 identified proteins in the analyzed vaccine charges. Obviously, at least a part of these proteins is immunogenic and non-toxigenic strains closely related to the vaccine producer strain are targeted by cross-reacting antibodies induced via proteins of the PW8 producer strain. More distantly related *C. diphtheriae* strains as well as *C. ulcerans* isolates showed less cross-reactivity with non-toxin proteins in Western blot experiments with human sera, an observation which may help to explain the population drifts in *C. diphtheriae* and the increasing number of *C. ulcerans* infections (see below) [25].

Although diphtheria toxoid vaccines were already introduced in the 1920s and consequently their safety track record is established over decades of diphtheria vaccination [26], side effects cannot be excluded and the cross-reactivity of diphtheria toxin-induced antibodies may affect diagnostic tests and cause harmful autoimmune reactions following vaccination [27]. When the sequence identity between the diphtheria toxin and the human proteome was analyzed at the pentapeptide level, only 31 pentapeptides were unique to the antigen, while the toxin shares 503 pentapeptides with the human proteome. Human proteins containing bacterial peptide matches include antigens linked to fundamental cellular functions, such as cell cycle control, proliferation, development, differentiation and neural functions [27,28]. In principle, these putative bacterial peptides can result in side effects depending on the immunogenicity. The authors concluded that the presented in silico proteomics approach offers a rational basis for the design of peptide-based vaccines based on the 31 unique pentapeptides that specifically target diphtheria toxin without a potential risk of side effects due to cross-reactivity with human proteins. Furthermore, they concluded that the method is suitable for other putative vaccines [27,28].

The effects of antigen identity may also explain observations made in the frame of the COVID-19 pandemic. In a recent study, the similarity of SARS-CoV-2 proteins to vaccines directed against diphtheria, tetanus and pertussis infections (DTP vaccine) was analyzed. Putative cross-reactive epitopes with SARS-CoV-2 spike protein were found and may explain why children were less affected by the pandemic. The authors hypothesized that routine DTP vaccinations of children may have elicited cross-reactive immunity and protection against SARS-CoV-2 infection [29].

### 1.2. Corynebacterium belfantii and Corynebacterium rouxii

Recently, a number of former *C. diphtheriae* strains of biovar Belfanti were taxonomically newly described as separate species, *Corynebacterium belfantii* [30] and *Corynebacterium rouxii* [31,32]. While partially included in the former in silico studies of *C. diphtheriae* mentioned above, no separate proteome studies were carried out for these new species to date, while limited information is available for mass spectrometry-based identification, as described below.

### 1.3. Corynebacterium ulcerans

*C. ulcerans* was first isolated almost one hundred years ago from a case of a diphtheria-like respiratory infection [33], but it can also cause skin and systemic infections in humans. The bacteria colonize a wide variety of different domestic and wild animals, either as commensals or as pathogens (for a review, see [34]). For a long time, human infections were rare and mainly restricted to populations in direct contact with domestic livestock and to consumers of raw milk and unpasteurized dairy products as *C. ulcerans* is an etiological agent of mastitis in dairy cattle [35,36]. However, during the last few decades, the frequency and severity of human infections by *C. ulcerans* have been increasing and in Western European countries, *C. ulcerans* is recognized as an emerging pathogen, which is outnumbering *C. diphtheriae* as the cause of diphtheria [37,38,39].

The virulence factors of this pathogen were not well-investigated and as a basis for pathogen–host interaction studies, surface-located proteins and the exoproteome of *C. ulcerans* strain 809, isolated from a fatal case of human respiratory tract infection, and BR-AD22, isolated from a nasal swap of an asymptomatic dog, were analyzed [40,41]. Cell surface proteins were isolated via tryptic shaving and proteins secreted into the medium were precipitated using trichloric acid [41]. After mass spectrometric analysis via nanoLC-MS/MS, an almost identical collection of virulence factors was detected in the culture supernatant and surface protein fractions of the two strains despite their isolation from different host organisms (human versus dog) and the different symptoms caused (fatal versus asymptomatic infection). In strain 809, 13 virulence-related proteins out of 14 pathogenicity-associated proteins identified earlier [40] were observed in the study: a putative ribosome-binding protein with high structural similarity to Shiga-like toxins, a CP40 protease precursor, phospholipase D, four fimbrial subunits, a resuscitation-promoting factor interacting protein, a cell wall-associated hydrolase, a sialidase precursor, two venom serine proteases and a trypsin-like protease. Only two of these, the putative ribosome-binding protein and venom serine protease 2A, were not observed in the secretome of the strain BR-AD22. The appearance of three and four different proteases, respectively, and the protease activity observed via casein zymography hint at proteolysis being the major pathogenicity mechanism. This is in accordance with the tissue destruction, which is characteristic of many *C. ulcerans* infections [41].

Unexpectedly, the study indicated that the canine isolate BR-AD22 is significantly less stable and less stress-resistant than the human isolate 809. During exponential growth, 38% of the predicted proteins encoded by the BR-AD22 genome were found, while only 17% of the proteins encoded in the genome sequence of strain 809 were detectable in the medium. The fact that a considerably high number of intracellular proteins are found at all is a rather rare observation in proteome studies of *Corynebacterium* species, since corynebacteria are typically very robust due to their complex cell wall structure [2,42,43] and significantly less proteins were found in the supernatant of other pathogenic corynebacteria in previous studies [10,44,45,46,47,48,49].

Besides a number of proteins with an annotated function, many functionally not annotated and hypothetical proteins were detected in this *C. ulcerans* proteome analysis, supporting the idea that proteomics may be a helpful tool for the basic characterization of protein expression and localization even in the case of a very basic genome annotation.

### 1.4. Corynebacterium silvaticum

*C. silvaticum* is a new species comprising bacteria formerly described as untypical *C. ulcerans* strains isolated from roe deer and wild boars in Germany and Austria [50]. Later, a broader distribution of the species with strains isolated in Portugal was reported [51]. A phylogenetic analysis showed that the species has diverged into two clades. Clade 1 is formed by toxigenic strains. In contrast, clade 2 contains non-toxigenic toxin gene-bearing (NTTB) strains, which cannot produce diphtheria toxin due to a frame shift in the *tox* gene or its promoter region [52].

While in principle less detrimental, even the NTTB strain W25 was cytotoxic to human epithelial cells and in the invertebrate model systems *Caenorhabditis elegans* and *Galleria mellonella* [53]. In the frame of a first proteome study of this species, the whole cell and cell surface fraction as well as the exoproteome of strain W25 were analyzed [54]. A total number of 1305 different proteins were detected, comprising 64.8% of the theoretical proteome of strain W25, when cells were grown in standard bacteriology medium. From the set of 15 virulence factors defined [55], 12 were identified in this study, namely phospholipase D, sialidase, a peptidoglycan endopeptidase, a cell wall peptidase, venom serin protease 2, a type VII secretion-associated serine protease, three proteins related to mycolic acid synthesis, a hydrolase and two resuscitation-promoting factors. The study indicated that a newly identified trypsin-like protease, formerly described as uncharacterized protein, seems to be a major virulence factor of *C. silvaticum* based on the fact that it is by far the most abundant secreted protein of this pathogen. In fact, 88.1% of the secreted proteome fraction was attributed to this protein [54]. Taken together, the proteome analysis *of C. silvaticum* W25 helped to functionally annotate uncharacterized proteins and indicated that proteolysis may be a major pathogenicity mechanism in this species, as in the case of *C. ulcerans*.

### 1.5. Corynebacterium pseudotuberculosis

Based on the highly effective mass vaccination strategy of the World Health Organization established in the 1970s, infections by *C. diphtheriae* became rare, although even today outbreaks are still reported, with a focus on countries with poor access to public health systems, e.g., Ethiopia, India, Indonesia, Madagascar, Nepal, Pakistan, Venezuela and Yemen [56]. In contrast, the commercially available vaccines for the zoonotic pathogen *C. pseudotuberculosis* are inefficient in providing total protection, have questionable safety levels as well as severe side effects and also effective drugs are lacking, despite the fact that the bacterium is causing significant economic losses.

The species is divided into two biovars based on the biochemical properties, infected host animals and evoked diseases. Biovar ovis is the causative agent of caseous lymphadenitis (CLA), a chronic contagious disease characterized by abscess formation in superficial lymph nodes and in subcutaneous tissues in small ruminants, especially sheep and goats. In addition, it can cause mastitis in dairy cattle [57,58]. Biovar equi is responsible for abscess formation as well as ulcerative lymphangitis in equines and edematous skin disease in buffalos [59]. Due to the negative economic impact of *C. pseudotuberculosis* infections, e.g., impaired wool, meat, milk and leather production, and the lack of efficient drugs, it is not astonishing that a considerably high number of proteomic studies was carried out for this zoonotic species.

As in the case of *C. diphtheriae*, *C. ulcerans* and *C. silvaticum*, a number of early studies focused on the protein inventory of *C. pseudotuberculosis.* Especially the exoproteome was analyzed based on the idea that this comprises the first pathogen proteins in contact with the host. A comparison was performed of the exoproteomes of different *C. pseudotuberculosis* strains isolated from goat and sheep. In the first of these studies, 49 out of 93 identified exoproteins observed in a gel-based proteome analysis were observed only in one of two strains, and the authors assumed that variations may account for the differential virulence of the investigated strains [45]. In a similar study, a further 11 new proteins were identified [48]. To look deeper at the complexity of the *C. pseudotuberculosis* exoproteome, two-dimensional difference gel electrophoresis (2D-DIGE) experiments were carried out, which also supported a differential exoproteome expression despite identical genome information [47]. Taken together, the authors of these initial research projects assumed that the newly identified proteins may play an important role in the physiology and virulence of *C. pseudotuberculosis* and that variations reflect different host adaptations [45,47,48].

In vitro studies also suggested an activation of the general stress response and a specific reaction to nitrosative stress as well as changes in the pathways involved in cellular metabolism, detoxification, transcriptional regulation and DNA synthesis and repair [49,60], most likely as an adaptation to the hostile environment in phagocytic cells. In these studies, extracellular proteins were extracted via the three-phase partitioning technique using ammonium sulfate and *n*-butanol addition and identified and based on a method of liquid chromatography–mass spectrometry acquisition (LC–MS). High-throughput proteome analyses of *C. pseudotuberculosis* wild-type and a deletion mutant of *sigE*, encoding the extracytoplasmic function sigma factor σ^E^, identified extracellular proteins induced by nitric oxide/peroxide stress and demonstrated the participation of σ^E^ in the composition of this bacterium’s exoproteome [60]. High-throughput proteomics also revealed 835 proteins, representing approximately 41% of the predicted proteome of *C. pseudotuberculosis* strain 1002, of which 102 proteins were exclusively found in nitric oxide-stressed bacteria and a further 58 proteins differentially regulated upon NO stress. Proteome data obtained indicate an activation of the redox and general stress responses as well as changes in the proteins involved in metabolic pathways, transcriptional regulation as well as and DNA synthesis and repair [49], similar to the situation in *C. diphtheriae* [17].

Changes in protein abundance were also observed in bacterial isolates from a natural host [61], and a shift in the virulence potential of *C. pseudotuberculosis* biovar ovis after passage in a murine host model was observed [62,63]. Comparative proteome analyses of the laboratory reference strain Cptb_C231 and three field strains isolated from the lymph nodes of infected sheep were carried out and a total of 1358 proteins were identified, leading to a proteome coverage of approximately 65%. While the majority of proteins had a similar abundance, some of the identified proteins showed differences in the field isolates compared to the laboratory strain. The field isolates were characterized via the induction of proteins related to hypoxia, starvation and thiopeptide biosynthesis [61].

Label-free proteome analyses of culture supernatants were carried for *C. pseudotuberculosis* 1002_ovis grown under standard laboratory conditions and after the re-isolation of bacteria from the spleen of infected mice. In comparison, 118 proteins were found to be differentially expressed. The major virulence factors of *C. pseudotuberculosis*, i.e., the CP40 protease and phospholipase D, were exclusively found after murine passage, in addition to other proteins involved in the detoxification, pathogenesis and protein secretion pathways [62]. Similar results were obtained with the *C. pseudotuberculosis* strain 258_equi after murine passage. Also in this case, the induction of proteins involved in bacterial pathogenesis, bacterial secretion systems and protein export pathways were differentially expressed in bacteria isolated from the spleen of infected mice [63].

When *C. pseudotuberculosis* was grown in bovine fetal serum, analysis of the membrane-associated proteome via LC-MS/MS revealed 22 proteins with pathogenic potential differentially expressed by *C. pseudotuberculosis*. Based on the results obtained, it was assumed that pathogenesis may be connected to iron and oligopeptide uptake, protein secretion, bacterial resistance and adhesion [64].

Besides the adaptation of metabolic and stress-related pathways to the host environment, biofilm formation is an important pathogenicity mechanism and some strains of *C. pseudotuberculosis* are forming biofilms already under laboratory conditions. In a comparative proteomic analysis of a biofilm-forming and a non-forming strain, cell wall synthesis and exopolysaccharide biosynthesis proteins were identified, which were either exclusively synthesized or upregulated in biofilm-forming *C. pseudotuberculosis* isolated from goats [65]. Biofilm-forming *C. pseudotuberculosis* CAPJ4 differentially expressed a penicillin-binding protein, which participates in the formation of peptidoglycans, and showed an increased expression of N-acetylmuramoyl-L-alanine amidase and galactose-1-phosphate uridylyltransferase, which are crucial for exopolysaccharide biosynthesis for biofilm formation [65].

While the approaches described above are of more general importance, a number of studies, especially from Brazilian research groups, were focusing on an integrative approach in order to discover putative targets for diagnosis and therapy, starting already with the availability of the first genome sequences. Advances in DNA sequencing techniques, analysis of *C. pseudotuberculosis* genome organization and software packages to analyze, e.g., pathogenicity islands or integrate RNA sequencing and proteome data were discussed [66]. These attempts resulted in a sophisticated workflow, which started from publicly available genome data to establish a core genome, which was filtered for differentially expressed genes, which were subsequently analyzed in respect of essentiality, protein interactions and antigenic properties. When genome and proteome data were combined with a modeling approach, a set of 10 essential *C. pseudotuberculosis* proteins was identified (Table 2). Four of these proteins had no host homologs in the putative host species, i.e., cow, horse, man and sheep, and thus qualified as putative drug targets. These were subjected to virtual screening of a drug-like compound library and for all the targets, putative drug molecules were found among the top-ranking compounds [67]. Further proteins in this respect and putative vaccine targets were identified in the frame of an integrated in silico study [68], an in silico study focusing on predicted antigenic epitopes [69] and a proteomics study combined with a reverse vaccinology approach [70] (Table 2). Today, most commercially available *C. pseudotuberculosis* vaccines are directed either against inactivated phospholipase D, inactivated cells or cell fractions, in addition to a few live-attenuated vaccines [71]. To the best of our knowledge, clinical studies based on the newly identified targets were not carried out.

In a complementary approach, immune-reactive exoproteins of two *C. pseudotuberculosis* strains were studied in a serological proteome analysis using blood sera from goats and sheep. With 13 immuno-reactive proteins identified from both strains, also this proteomic approach revealed putative targets for vaccine development [72]. Six of the identified proteins from the core immune-proteome were of unknown functions, surface layer protein A, cell-surface hemin receptor HtaA and two trehalose corynomycolyl transferases were related to cell envelope functions and resuscitation-promoting factor plays a role in the stress response and virulence. In addition, neuraminidase (sialidase) and invasion-associated protein p60 are part of the accessory seroproteome and putatively related to the cell surface and virulence [72].

Recently also a host proteome was addressed to unravel the molecular patterns and immune response mechanisms induced by *C. pseudotuberculosis*. When the spleen proteome of infected dairy goats was analyzed using the KEGG database, a total of 102 pathways were significantly enriched, including the lysosome, phagosome and mineral absorption pathways. The results obtained indicated that *C. pseudotuberculosis* infection can impair immune response mechanisms and induce immune cell death [73]. Further proteome analyses of the spleen of dairy goats infected with *C. pseudotuberculosis* for different time periods indicated the adaptation of the host animals to the bacteria [74].

## 2. Proteomics as a Diagnostic Tool

Matrix-assisted laser desorption/ionization time-of-flight mass spectrometry (MALDI-TOF MS) has been used as a fast and precise tool for the identification of bacteria, including corynebacteria. With this approach, mass spectra of tested organisms are compared to reference spectra in the databases to find the closest match. Before the introduction of proteomics, detection methods for the diagnosis of diphtheria and identification of potentially toxigenic corynebacteria were traditionally relying on a combination of basic species identification, biochemical differentiation approaches and molecular differentiation methods [75,76]. The criteria monitored included growth on selective media, colony color and form, hemolytic properties and a set of biochemical reactions (API Coryne) combined with fatty acid profile analysis and 16SrDNA sequencing as the gold standard. In addition, the expression of the diphtheria toxin was validated via real-time *tox* PCR and the Elek test [75,76]. In contrast to these time-consuming methods, an identification result from a single bacterial colony can be obtained via MALDI-TOF MS in less than 15 min. This is an immense advantage of proteomics, since especially in the case of potentially toxigenic *Corynebacterium* species, a rapid identification is crucial for appropriate antitoxin and antibiotic treatment. Already in 2010, a collection of 116 *Corynebacterium* strains from 18 species were examined via MALDI-TOF MS. All 90 potentially toxigenic *C. diphtheriae*, *C. ulcerans* and *C. pseudotuberculosis* strains were correctly identified by means of MALDI-TOF MS [77]. With improved databases, MALDI-TOF MS analysis showed superior performance compared to the identification of corynebacteria and is the backbone of laboratory diagnosis now [76].

A current limitation of the method in respect to *C. diphtheriae* strains is the correct identification of the newly described species *C. belfantii* and *C. rouxii*. The MALDI-TOF spectra of the *C. belfantii* strains were indistinguishable from those of *C. diphtheriae* biovar mitis and gravis [30]. However, at least the already described specific spectral peaks may allow the standard identification of *C. rouxii* also by means of MALDI-TOF MS in combination with commercially available databases in the near future [31,32]. A mass spectrometric demarcation was also achieved for the newly described *C. silvaticum* [78] and as shown recently, in case of *C. pseudotuberculosis*, MALDI-TOF MS can contribute not only to the identification at species level but can additionally support biovar differentiation [79,80].

## 3. Development, Limitations and Perspectives

The first proteome analyses of toxigenic corynebacterial carried out relied on two-dimensional gel electrophoresis followed by tryptic digest and peptide mass fingerprint analysis. The higher sensitivity of gel-free protein separation methods and superior mass spectrometry methods allowed faster and more reliable identification of increasing numbers of distinct proteins and the quick analysis of new isolates. While many genome sequences of toxigenic bacteria are available today, the depth of analysis is widely differing. For example, from 431 genome sequences available for *C. diphtheriae* in the NCBI genome database, only 68 are complete [81], which is obviously a drawback affecting proteomics studies. A better quality or the curation of existing data may help to improve this situation.

An aspect widely neglected in proteome studies of toxigenic corynebacteria is the analysis of proteoforms, which may result from sequence variations or post-translational modifications in the case of bacteria, where RNA splicing processes are not found [82]. A recent manuscript provided first data on the role of the post-translational S-mycothiolation in redox control in *C. diphtheriae* [17]. However, protein characterization at the proteoform level may be of crucial importance to fully understand, e.g., the signaling processes involved in host–pathogen interaction, since distinct proteoforms may be responsible for specific biological functions. A precise determination of the amino acid sequence, nature and localization of post-translational modifications can be achieved using different strategies, either starting from the peptide level (bottom-up proteomics, BUP) or from the level of intact proteins (top-down proteomics, TDP), and a combination of bottom-up and top-down approaches may improve the quality of the proteoform assessment [83]. Corresponding methods for the visualization of BUP and TDP were presented and discussed recently [84]. Most interesting would be a combined proteoform analysis of toxigenic corynebacteria and their host cells to elucidate the interaction of the pathogen and host signaling pathways. Recently, we started a combined and time-dependent transcriptome and proteome analysis of *C. diphtheriae*–macrophage interaction as a basis for a modeling approach concerning the involved signaling pathways, which is in progress now.

## 4. Conclusions

Proteome studies of toxigenic corynebacteria show impressively that functional genomics studies are crucial to complement genome information. Especially the combination of MS-based proteomics with bioinformatic tools helped significantly to elucidate the functional aspects of corynebacterial genomes and to handle the genome and proteome complexity. The combination of proteomic and bioinformatic approaches has been a highly fruitful strategy to study proteome responses to environmental changes or host cell contact, and based on the technical advances in mass spectrometry, more complex samples from naturally infected host tissue material may be analyzed via proteomics, leading to a deeper understanding of the infection process. In addition, proteome studies have been used to discover new vaccine and drug targets, as especially exemplified for *C. diphtheriae* and *C. pseudotuberculosis* [21,22,55,67,70,85].

## Figures and Tables

**Table 1:** Proteomics-directed identification of vaccine and drug targets in *C. diphtheriae*.

Protein	In Silico Identification	In Vitro Identification
BioB	[21]	[23]
DIP0983	[21]	[23]
DIP1084	[21]	
GlpX, fructose 1,6-bisphosphatase II	[21,22]	[23]
HisE, phosphoribosyl-ATP pyrophosphatase	[21,22]	
NusB	[21]	[23]
RpsH, 30S ribosomal protein S8	[21,22]	[23]
SmpB	[21]	[23]

**Table 2:** Proteomics-directed identification of vaccine and drug targets in *C. pseudotuberculosis*.

Protein	Host Homolog Identified	In Silico Identification	In Vitro Protein Detection
Adk, adenylate kinase	yes	[67]	
AspA, aspartate ammonia lyase	yes	[67]	
CopC, copper resistance protein	n.d. *	[68]	
CP0126a, hypothetical protein	n.d. *	[69]	
CP0369, phosphoesterase PA-phosphatase related protein	n.d. *	[69]	
CP1957, CmtB, trehalose corynomycolyl transferase B	n.d. *	[69]	
FtsI, penicillin-binding protein	n.d. *	[68]	[70]
FumC, class II fumarate hydratase	yes	[67]	
GlyA, hydroxymethyltransferase	yes	[67]	
Gnd, 6-phosphogluconate dehydrogenase	yes	[67]	
IspH, 4-hydroxy-3-methylbut-2-enyl diphosphate reductase	no	[67]	
MtrA, DNA-binding response regulator	no	[67]	
MtrB, sensor histidine kinase	n.d. *	[68]	
Ndh, NADH dehydrogenase	n.d. *	[68]	[70]
NrdI, ribonucleoside-diphosphate reductase alpha chain	no	[67]	
SenX3, signal transduction histidine kinase	n.d. *	[68]	[70]
TcsR, two-component system transcriptional regulator protein	no	[67]	
YkuE, metallophosphoesterase	n.d. *	[68]	

* not determined.

## Data Availability

Data sharing is not applicable to this article.
